# Errors in Tables

**DOI:** 10.1001/jamanetworkopen.2025.60466

**Published:** 2026-01-26

In the Consensus Statement titled “Rationale and Methodological Approach Underlying the Development of the Sequential Organ Failure Assessment (SOFA)–2 Score: A Consensus Statement,”^1^ published October 29, 2025, there were errors in Tables 3 and 4. In Table 3, footnote f incorrectly applied the unit mm Hg to ratio data. In Table 4, the renal organ system was incorrectly listed as the kidney organ system. This article has been corrected.^1^
